# Supplementary material for: Association between Intracellular Calcium Signaling and Tumor Recurrence in Human Non-Functioning Pituitary Adenomas
Source: Int J Mol Sci. 2024 Apr 3;25(7):3968. doi: 10.3390/ijms25073968 (PMC11011867; doi:10.3390/ijms25073968)
Supplement: Supplementary file 1 [file ijms-25-03968-s001.zip › ijms-2909217-supplementary.pdf]

**Supplementary Table S1.** CNFPA cells classification according to their $Ca^{2+}_i$ activity and response to hypothalamic secretagogues.

| Tumor recurrency | Subject | Spontaneous activity of $Ca^{2+}_i$ | Proportion (%) of | | | Mean fluorescence per stimulus (dF/Fmin) | | | | | | |
|---|---|---|---|---|---|---|---|---|---|---|---|---|
| | | | Non-responsive cells | Mono-responsive cells | Multi-responsive cells | Spontaneous activity of $Ca^{2+}_i$ | GHRH | CRH | GnRH | TRH | TRH-DA | High potassium |
| Not recurrent | 4 | 0 | 97.9 | 1.8 | 0.2 | 1.004137 | 1.003880 | 1.003719 | 1.005468 | 1.004417 | 1.004130 | 1.070270 |
| | 7 | 0 | 97.8 | 1.4 | 0.7 | 1.002690 | 1.003719 | 1.005459 | 1.003259 | 1.004114 | 1.003449 | 1.010516 |
| | 11 | 0.16 | 86.9 | 10.5 | 2.5 | 1.001322 | 1.001876 | 1.002592 | 1.003140 | 1.006339 | 1.001651 | 1.048222 |
| | 3 | 2.8 | 76 | 12.4 | 11.6 | 1.003853 | 1.007563 | 1.008806 | 1.009810 | 1.008582 | 1.017288 | 1.293667 |
| | 9 | 0 | 75 | 13.6 | 9.8 | 1.001774 | 1.002056 | 1.002418 | 1.002276 | 1.004268 | 1.002413 | 1.033359 |
| | 10 | 6.4 | 61.7 | 24.8 | 13.4 | 1.003924 | 1.001598 | 1.003465 | 1.007469 | 1.004067 | 1.001645 | 1.030439 |
| | 1 | 12.4 | 56 | 24.9 | 18.9 | 1.008477 | 1.012457 | 1.011489 | 1.010444 | 1.009485 | 1.022113 | 1.153025 |
| | 8 | 0 | 3.16 | 96.3 | 0.22 | 1.005131 | 1.006502 | 1.005109 | 1.004617 | 1.006326 | 1.006214 | 1.017703 |
| Recurrent | 6 | 5.2 | 12.5 | 54.9 | 32.5 | 1.001691 | 1.003667 | 1.001983 | 1.003587 | 1.001846 | 1.001783 | 1.076448 |
| | 2 | 57.9 | 26.9 | 15.9 | 57.1 | 1.008586 | 1.008516 | 1.005542 | 1.011239 | 1.010955 | 1.017874 | 1.040070 |
| | 12 | 32.7 | 50.6 | 20.9 | 28.5 | 1.001077 | 1.001259 | 1.001246 | 1.002078 | 1.001625 | 1.001127 | 1.056638 |
| | 5 | 11.7 | 42.3 | 29.4 | 28.2 | 1.003405 | 1.002714 | 1.005640 | 1.002659 | 1.002614 | 1.003185 | 1.005360 |
| | 13 | 32.3 | 55 | 18.3 | 26.5 | 1.000868 | 1.000940 | 1.001087 | 1.001005 | 1.001121 | 1.001011 | 1.009333 |
